# Three-Dimensional Core-Branch α-Fe_2_O_3_@NiO/Carbon Cloth Heterostructured Electrodes for Flexible Supercapacitors

**DOI:** 10.3389/fchem.2019.00887

**Published:** 2020-01-08

**Authors:** Miao Zhang, Xifei Li, Xiaohua Wang, Dejun Li, Naiqin Zhao

**Affiliations:** ^1^Tianjin International Joint Research Centre of Surface Technology for Energy Storage Materials, School of Physics and Materials Science, Tianjin Normal University, Tianjin, China; ^2^School of Materials Science and Engineering and Tianjin Key Laboratory of Composite and Functional Materials, Tianjin University, Tianjin, China

**Keywords:** flexible supercapacitors, nanorods arrays, core-branch, carbon cloth, electrochemical performance

## Abstract

A convenient and scalable hydrothermal method was developed for the fabrication of the core-branch Fe_2_O_3_@NiO nanorods arrays directly grown on flexible carbon cloth (denoted as Fe_2_O_3_@NiO/CC). Such a unique architecture was applied as an electrode of the supercapacitors. As a result, the Fe_2_O_3_@NiO/CC exhibited a high areal capacitance ~800 mF cm^−2^ at 10 mA cm^−2^, which was about 10 times increase with respect to Fe_2_O_3_ nanorods array grown on carbon cloth (Fe_2_O_3_/CC). The Fe_2_O_3_@NiO/CC also had the long life cycle (96.8 % capacitance retention after 16,000 cycles) and remarkable rate capability (44.0 % capacitance loss at a very large current density of 100 mA cm^−2^). The superior performance of the Fe_2_O_3_@NiO/CC should be ascribed to the reduction of the contact resistance and the free-standing structure of the flexible electrode. This study provides a novel strategy to construct high-performance flexible electrode materials with unique core-branch structure by incorporating two different pseudocapacitive materials.

## Introduction

With the growing needs of power and energy for the portable devices, electric vehicles and portable electronics, more and more attention has been focused on the advanced energy storage devices (Xia et al., 2018; Li J. M. et al., 2019). Among them, supercapacitors, also called as electrochemical capacitors (ECs), have been regarded as a new type of alternative energy resources, combining the advantages of the lithium ion batteries and the traditional capacitors (Ke and Wang, 2016; Xu et al., 2016). Supercapacitors have become the candidates for the high-performance power devices, because of the fast charging and discharging, high power density, long cycle life and the superior electrochemical stability (Yang W. et al., 2018). The property of the supercapacitors strongly depends on the characteristics of the electrode materials. At present, the main electrode materials of the supercapacitors are carbon materials, metal oxides/hydroxides, and conducting polymers (Geng et al., 2018). Carbon materials store energy via the electric double layer capacitance, whose capacitance is relatively low. On the other hand, the metal oxides/hydroxides and the conducting polymers store energy via the fast faradic pseudocapacitance, which achieve relatively high capacitance and high energy density (Liu et al., 2016; Savjani et al., 2016; Zhu et al., 2017). Therefore, the pseudocapacitive materials have been attracted more attention in the application in supercapacitors.

Hematite (α-Fe_2_O_3_) is one of the most promising candidates due to its high theoretical specific capacitance, suitable working window, low cost, abundance, and environmental benignity (Quan et al., 2016). However, the practical implementation of Fe_2_O_3_ is prevented by the poor electrical conductivity (~10^−14^ S cm^−1^) (Lee et al., 2012). To improve the electrochemical performance of the Fe_2_O_3_, many works have been done to achieve different nanostructured Fe_2_O_3_, such as nanoparticals, nanorods, nanowires, and nanoflowers (Reddy et al., 2010; Binitha et al., 2013; Zheng et al., 2016). The strategy can improve the available surface area and shorten the transmission paths of ions and electrons. Other researchers alternatively incorporate Fe_2_O_3_ with highly conductive carbon materials (mesoporous carbons, carbon nanotubes, carbon fibers, carbon sheets, etc.) or conducting polymers to improve the electrical conductivity (Hu et al., 2015; Raut and Sankapal, 2016). Most above materials are powder-like in macrostate, which have to be applied in supercapacitors by traditional assembly processes. However, the traditional assembly processes require polymer binder and additive, which increase the contact resistance and reduce the rate capability because of the electrochemical inactivity and insulativity (Yang W. et al., 2013; Zhang et al., 2017).

The binder-free material with the free-standing structure which can effectively simplify the preparation process of the electrodes and improve the electrochemical property of the supercapacitors (Li M. et al., 2015). Directly anchored on the current collector, the active materials can achieve outstanding performance, including remarkable rate capability and long life cycle stability, owing to the great electrode-electrolyte contact, the short ion/electron transport paths and the low contact resistance. More than that, a relatively low weight fraction of faradaic pseudocapacitive material is considered to achieve better rate performance and longer life cycles at the sacrifice of energy density in most cases (Fischer et al., 2007). Therefore, many efforts have been made for pseudocapacitive materials such as mixed oxides and binary metal oxide/hydroxides to increase the energy density (Li H. et al., 2015; Tian et al., 2017). The core-shell nanostructure is usually fabricated to incorporation two different metal oxides. But the homogeneous shell prohibits the ion penetration into the core region, leading to the core cannot realize electroactivity and reducing the speed of electron transfer. Due to the lack of well-defined microstructures and the poor contact between the mixed oxides, electrochemical performance is always not satisfactory. The big challenge is how to set up an integrated structure, in which both of metal oxides are excellent pseudocapacitive materials (Fe_2_O_3_, NiO, Co_3_O_4_, and MnO_2_), and the structural features and electrochemical property of each component are fully used, as well as the fast ion/ electron transmission path can be achieved (Kim et al., 2017).

Herein, we synthesize a simple and scalable approach by building a hybrid metal oxides core/branch nanorods arrays for supercapacitors. The NiO possessing relatively low electrical resistance and high theoretical specific capacitance is decided as branch. The core-branch Fe_2_O_3_@NiO nanorods arrays are directly grown on flexible carbon cloth (denoted as Fe_2_O_3_@NiO/CC) as high-performance flexible binder-free ECs. Avoiding the use of polymer binder and conductive additives, the carbon cloth acts as current collector providing more active sites and reducing the resistance. The Fe_2_O_3_ and NiO are excellent pseudocapacitive metal oxides with earth-abundant, easily available, very cheap, and environment friendly. The core-branch structure can contribute to the energy storage. Different from core-shell structure, the core-branch is more beneficial to the ion penetration into the core region and realizes each constituent effectively utilized. Moreover, the unique core-branch structure can provide a large reaction interface, improve the charge transportation and obtain the high electrical conductivity of the electrodes, and supply more channels to increase the diffusion path of the electrolyte ions. The growth mechanism of this structure has been further analyzed according to the experiment. The electrochemical performance of Fe_2_O_3_@NiO/CC as the electrode materials in ECs has been investigated as well. This research provides a promising measure to design and prepare the hybrid metal oxides anodes with the improved electrochemical performance, which is hopeful for the application in energy storage/conversion devices.

## Experiment Section

### Materials

Carbon cloth was purchased from Alfa Aesar China (Tianjin) Co. Ltd., FeCl_3_, Na_2_SO_4_, HNO_3_, Ni(NO_3_)_2_·6H_2_O and urea were purchased from Tianjin Chemical Reagent Company (Tianjin, China). Reagents were of analytical grade and used without further purification process.

### Synthesis

Typically, carbon cloth was ultrasonically cleaned in 32% HNO_3_, ethanol and deionized water in sequence and dried under the atmospheric condition. FeCl_3_ (0.32 g), Na_2_SO_4_ (0.28 g) were dissolved into deionized water (40 mL) by vigorous stirring about 30 min to form a homogeneous solution, and then transferred to 50 ml Teflon-lined autoclave. A piece of carbon cloth (3 ^*^ 5 cm) was immersed into the prepared solution, then sealed and maintained at 120°C for 8 h. After the hydrothermal reaction, the carbon cloth was taken out and washed by deionized water and ethanol and then dried in air. Then, the production was soaked into 30 ml of aqueous solution containing 3.57 g Ni(NO_3_)_2_·6H_2_O and 0.45 g urea, which was stirred for 30 min, and transferred to 50 mL Teflon-lined autoclave. After heating 90°C for different time, simple was taken out and washed with deionized water and ethanol and then dried in air. Finally, the three-dimensional (3D) core-branch Fe_2_O_3_@NiO/CC was obtained through annealing in air 400°C for 1 h.

### Structural Characterization

The morphologies and characteristics of the products were characterized by the field emission scanning electron microscope (SEM, HITACHI S4800) and the high-resolution transmission electron microscope (TEM, JEOLJEM-2100f). The crystal structures of the samples were investigated by X-ray diffraction (XRD, Bruker D8 Advanced). The Brunauer–Emmett–Teller (BET) surface area and the pore size distribution were measured by the nitrogen adsorption isotherms with the autosorbiQ instrument (Quantachrome, U.S.).

### Electrochemical Test

All electrochemical measurements were carried out with a CHI660D electrochemical workstation in a conventional three-electrode mode in 3 M KOH aqueous solution as the electrolyte. The Fe_2_O_3_@NiO/CC, platinum, and Hg/HgO electrode were used as the working electrode, counter and reference electrode, respectively. The loading amount of Fe_2_O_3_@NiO for electrochemical testing is about 10.0 wt%. Cyclic voltammetry (CV) was investigated at various scan rates of 5, 10, 50, 100, 200 mV s^−1^, galvanostatic charge/discharge (GCD) measurements was employed at the current density of 5, 10, 20, 50, 100 mA cm^−2^, and electrochemical impedance spectroscopy (EIS) was conducted under a frequency from 10^5^ to 0.01 Hz. The areal specific capacitance measured by chronopotentiometry was calculated according to the equation as follows:

$$\begin{array}{l} C_{a}=I^{*}\Delta t/\left(s^{*}\Delta V\right) \end{array}$$

Where *I* is the constant discharging current, Δ*t* is the discharging time, Δ*V* is the voltage change excluding IR drop at a constant discharge current and *s* is the effective electrode area.

The structure and the electrochemical performances of carbon cloth and the single Fe_2_O_3_ nanorods grow on carbon cloth (denoted as Fe_2_O_3_/CC) also were tested.

## Results and Discussion

**Figure 1:**
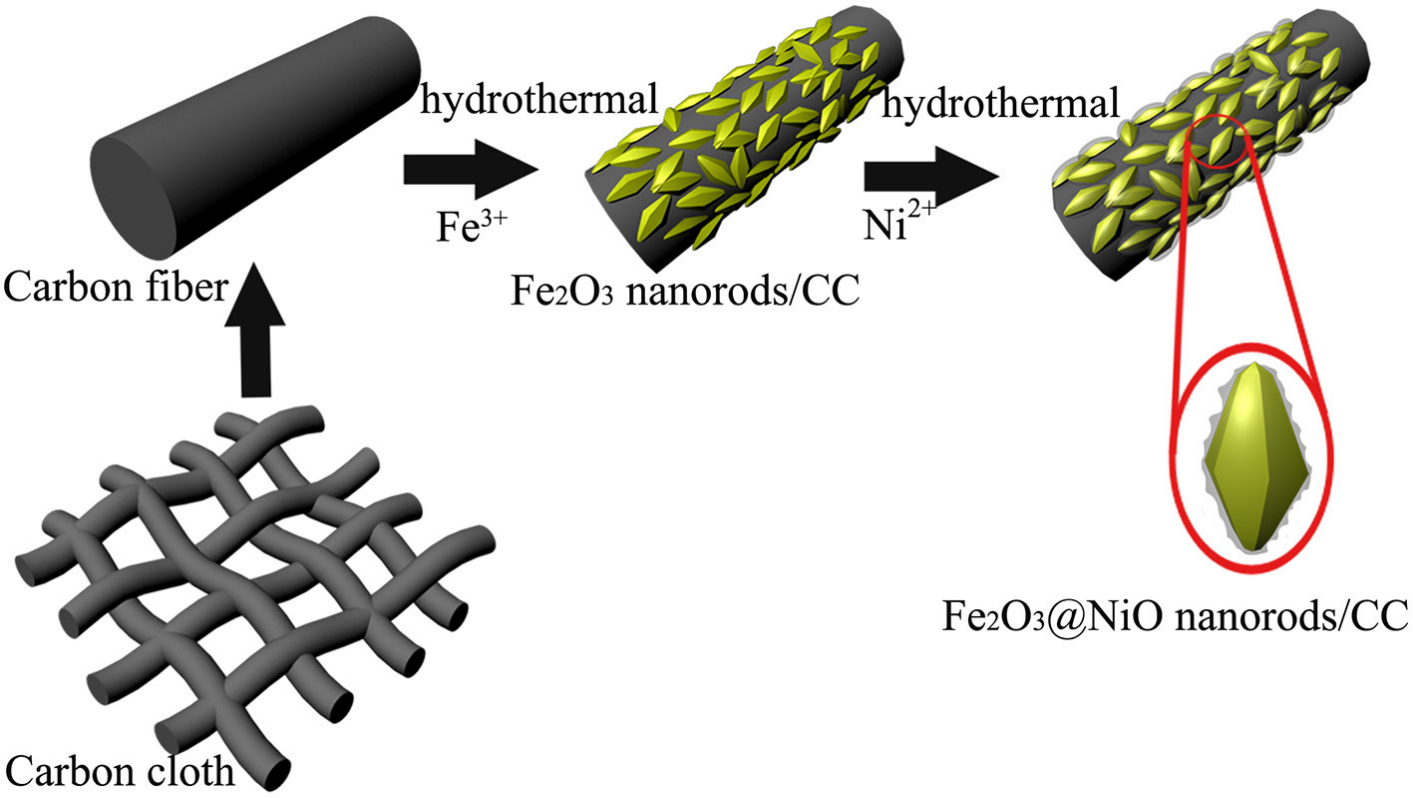
The schematic diagram of the growth process for Fe_2_O_3_@NiO/CC.

Carbon cloth with 3D textile structure and the diameter of 8-10 μm (Figure S1) serves as a scaffold for the composite architecture. With good flexibility and conductivity, carbon cloth directly acts as the current collector for the deposition of active material. As illustrated in Figure 1, 3D core-branch Fe_2_O_3_@NiO nanorods are synthesized on the carbon cloth through a process with simple two steps. Firstly, the Fe_2_O_3_ nanorods arrays are grown from carbon cloth substrate via the simple hydrothermal process, possessing many functional groups and improving their electrochemical activity. Secondly, the obtained porous nanorods are impregnated in the aqueous solution of Ni^2+^ and followed by the post-annealing in air, leading to the branch-like layer of NiO on the surface of the Fe_2_O_3_ nanorods. The bending state of the Fe_2_O_3_@NiO/CC has been shown in Figure S2, which proves the flexibility of Fe_2_O_3_@NiO/CC is excellent.

**Figure 2:**
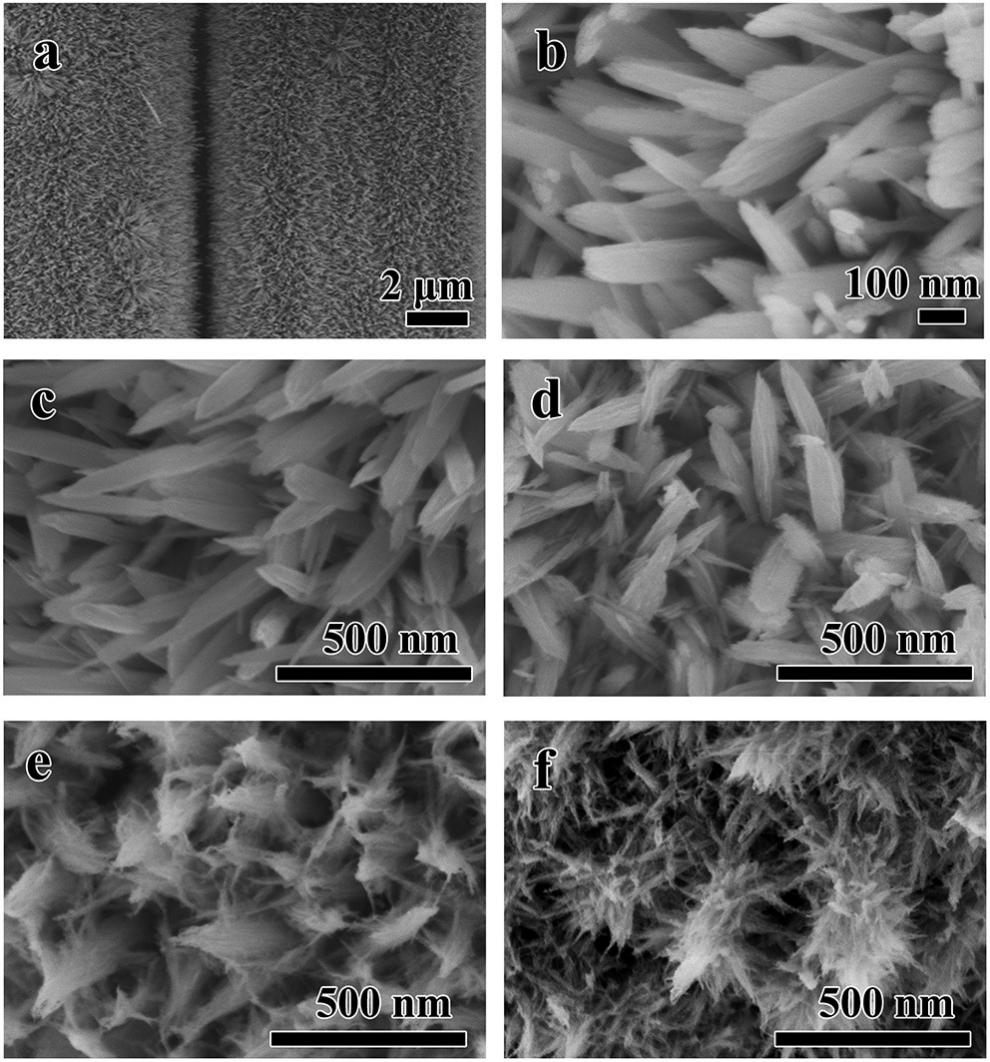
The typical SEM images of **(a,b)** the Fe_2_O_3_ nanorods/CC obtained after the first hydrothermal process and the Fe_2_O_3_@NiO/CC with different hydrothermal time during the second hydrothermal process **(c)** 3 h, **(d)** 6 h, **(e)** 9 h, **(f)** 18 h.

As shown in Figures 2a,b, the Fe_2_O_3_ nanorods with the diameter and length about 60~80 and 400~600 nm are grown homogeneously on the surface of the carbon cloth after first hydrothermal process. And then, the obtained nanorods work as the substrate for the subsequent growth of branch-like NiO through hydrothermal method. Figures 2c–f displays the typical SEM images of the samples during different hydrothermal time, which demonstrate the important roles of the reaction time in the hydrothermal reaction. Gradually, crystal growth of the NiO nanobranches based on the nucleation takes place along the easiest direction of the crystallization. Compared with the precursor, there is no obvious change after 3 h (Figure 2c), but the nanorods turn to be rough on the surface at 6 h (Figure 2d). When the reaction time is extended to 9 h, the NiO nanobranches grow along vertical direction without collapsing and cracking (Figure 2e). Dense NiO nanobranches are twined around every Fe_2_O_3_ nanorods uniformly with the forming of stable protective shell after 18 h hydrothermal time as displayed in Figure 2f.

**Figure 3:**
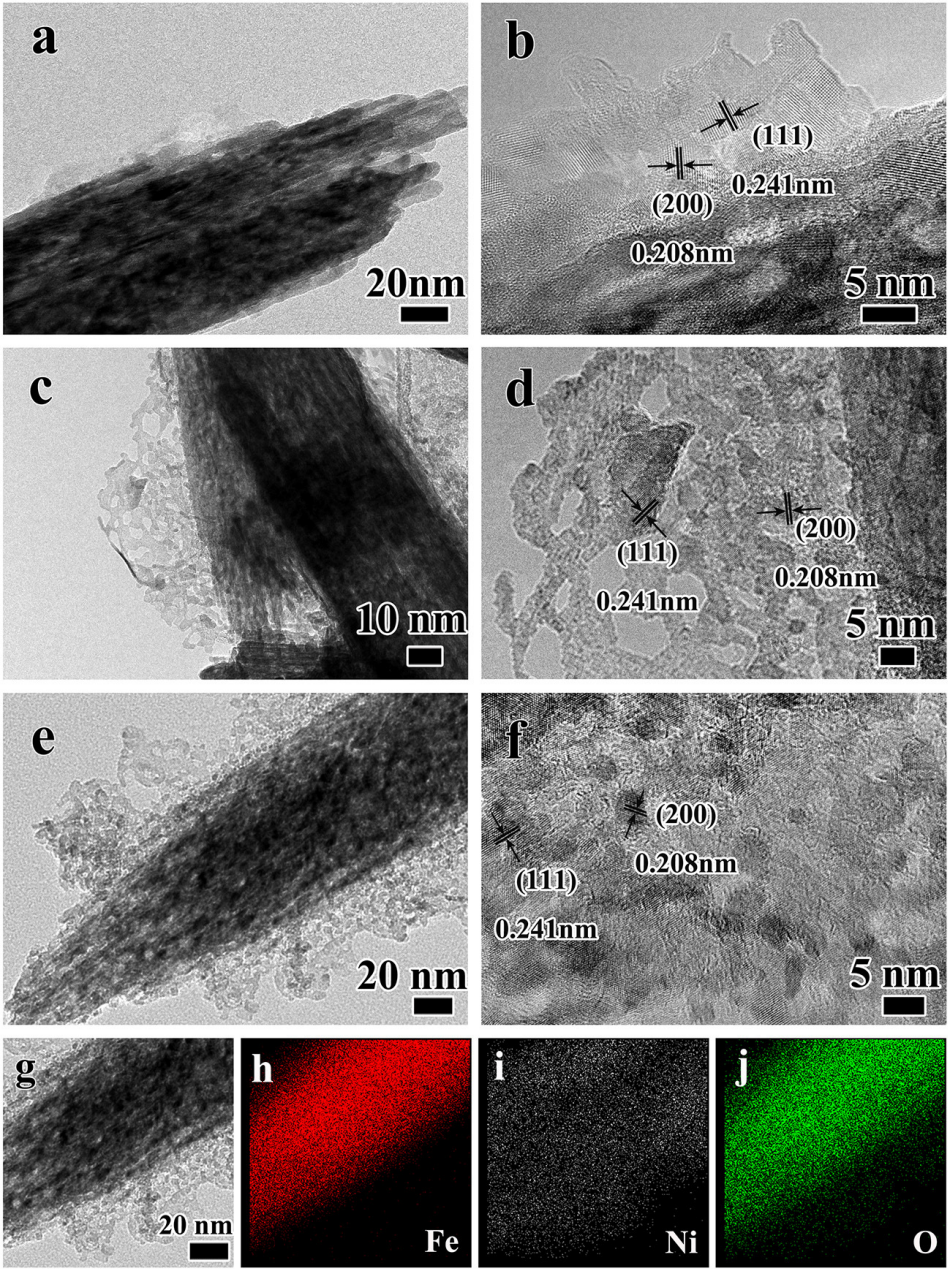
The TEM images of Fe_2_O_3_@NiO/CC at different hydrothermal time **(a,b)** 3 h, **(c,d)** 6 h, **(e,f)** 9 h, **(g)** the selected region TEM images of Fe_2_O_3_@NiO and the mapping analysis of Fe_2_O_3_@NiO, in which **(h)** the red one is Fe, **(i)** the white one is Ni and **(j)** the green one is O.

In order to further explore the structure, the core-branch structure of the Fe_2_O_3_@NiO on carbon cloth was further confirmed by TEM, as shown in Figure 3. Figure 3a reveals the formed NiO nuclei attaches to the surface of the Fe_2_O_3_ nanorods. Due to the hydrothermal time only 3 h, NiO nanobranches are unhomogeneous with the height about 10 nm. Figure 3b is the high-reslution image of the nanobranch structure, which showed the distances of crystal planes were 0.208 nm and 0.241 nm respectively, corresponding to the (200) and (111) crystal planes of NiO. Sequentially, crystal growth of the NiO nanobranches based on the nucleation takes place along the easiest direction of the crystallization with the height increasing to 50 nm in Figure 3c. The porous structure of the nanobranch NiO is because of the dehydration and contraction during the annealing process (Figure 3d). The height of the nanobranches increases with an increase in the time of hydrothermal process. After 9 h reaction time, there are dense branches attach to the surface of Fe_2_O_3_ nanorods with the excellent crystallinity (Figures 3e,f). In order to further analyze of the core-branch structure, the Energy-dispersive X-ray spectrometry (EDS) mapping analysis was used, the result was shown in Figures 3g–i. A single heterostructure unambiguously confirms the Fe_2_O_3_ core/NiO branch hybrid structure. Specifically, the Fe and O elements are mainly located at the nanorods region and the Ni is a fairly homogeneous and unapparent distribution due to the ultrathin nanostructure.

According to the experiment, during the hydrothermal process, the NiO nucleated and grew gradually along the direction perpendicular to the Fe_2_O_3_ nanorods with the extension of time, eventually forming the dendritic structure and loading on the surface of Fe_2_O_3_ uniformly. Generally, the crystal growth of NiO can be divided into two steps: initial nucleation and crystal growth (Zhang et al., 2010). At the initial stage with the high temperature and pressure, the urea hydrolyzed and NiO formed on the surface of Fe_2_O_3_ gradually. With the extension of reaction time, the NiO microcrystalline core continued to grow selectively along the direction of <100>. With the length increased, the small nanobranch structure formed and then the dendritic structure obtained.

**Figure 4:**
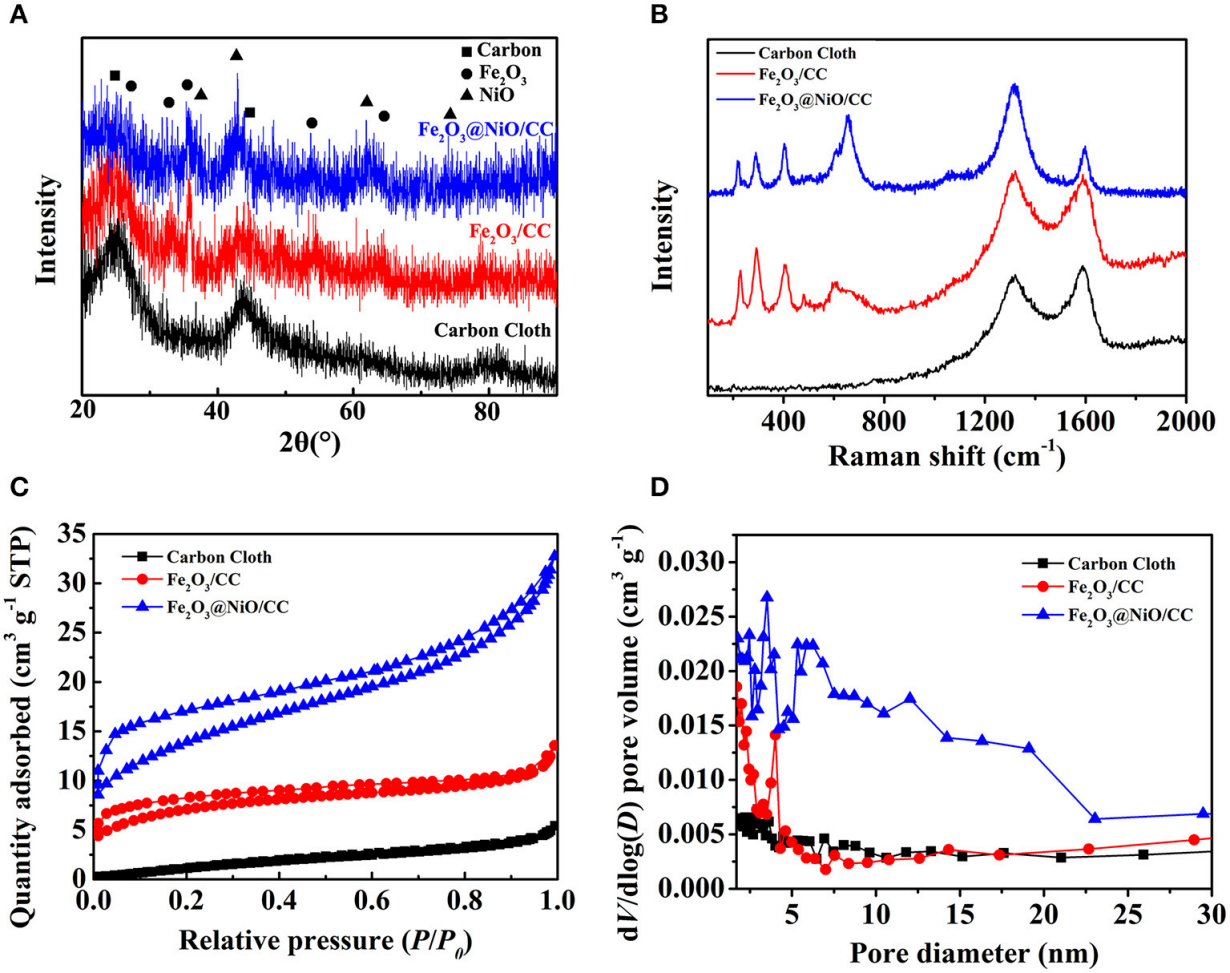
**(A)** The XRD patterns, **(B)** the Raman spectrum, **(C)** the N_2_ adsorption-desorption and **(D)** the pore size distributions by BJH method of carbon cloth, Fe_2_O_3_/CC and Fe_2_O_3_@NiO/CC.

To further understand the materials, the XRD was applied to analyze the elemental relative composition of the samples (Figure 4A). As the comparison, the XRD pattern of the carbon cloth is shown by the black line. Apart from the strong diffraction peak of carbon cloth, other diffraction peaks in the XRD pattern of α-Fe_2_O_3_ nanorods (red line) can be corresponded with the standard phase of hematite (JCPDS No. 33-0664). XRD pattern of Fe_2_O_3_@NiO/CC (blue line) reveals some new diffraction peaks at 37.25°, 43.28°, 62.87°, and 75.41° appear corresponding to (111), (100), (220), and (311) planes of NiO (JCPDS No. 47-1049), respectively. On the other hand, the Raman spectra had been conducted to characterize the structure and chemical composition of the materials (Figure 4B). The Raman peaks at 1,325 and 1,580 cm^−1^ are corresponded with the D band and G band of carbon materials (Zhang et al., 2017), which is contributed from the carbon cloth. The Raman peaks at about 229, 292, 403, and 1,300 cm^−1^ belong to the typical Fe_2_O_3_, which can be clearly observed, showing the homogeneous distribution of the Fe_2_O_3_ nanorods (Dong et al., 2015; Lu et al., 2015). Moreover, the Raman peaks at 381, 545, and 766 cm^−1^ are contributed by the NiO in Fe_2_O_3_@NiO/CC. The uniform distribution of Fe_2_O_3_@NiO on carbon cloth is important for the electrochemical property of the electrode. Furthermore, the specific surface area and the pore size distribution of the core-branch structure are also significant to promote the high-performance supercapacitors. The N_2_ adsorption isotherm is conducted in Figure 4C showing the typical II isotherm. The BET surface area of Fe_2_O_3_@NiO/CC and carbon cloth of is 23 and 6.71 m^2^g^−1^, indicating the increase of porous structures in the composite. It indicates that the specific surface area dramatically influenced by the content of Fe_2_O_3_@NiO. The Barrett-Joyner-Halenda (BJH) pore size distributions of the samples are as shown in Figure 4D. Most of the pores are distributed in the mesoporous range, the nanoporous range at 3, 4, and 5 nm, as well as the mesoporous range between 10 and 20 nm. The multistage pore structure may be favorable for the access of the electrolyte through the pore channels to increase effective contact area.

**Figure 5:**
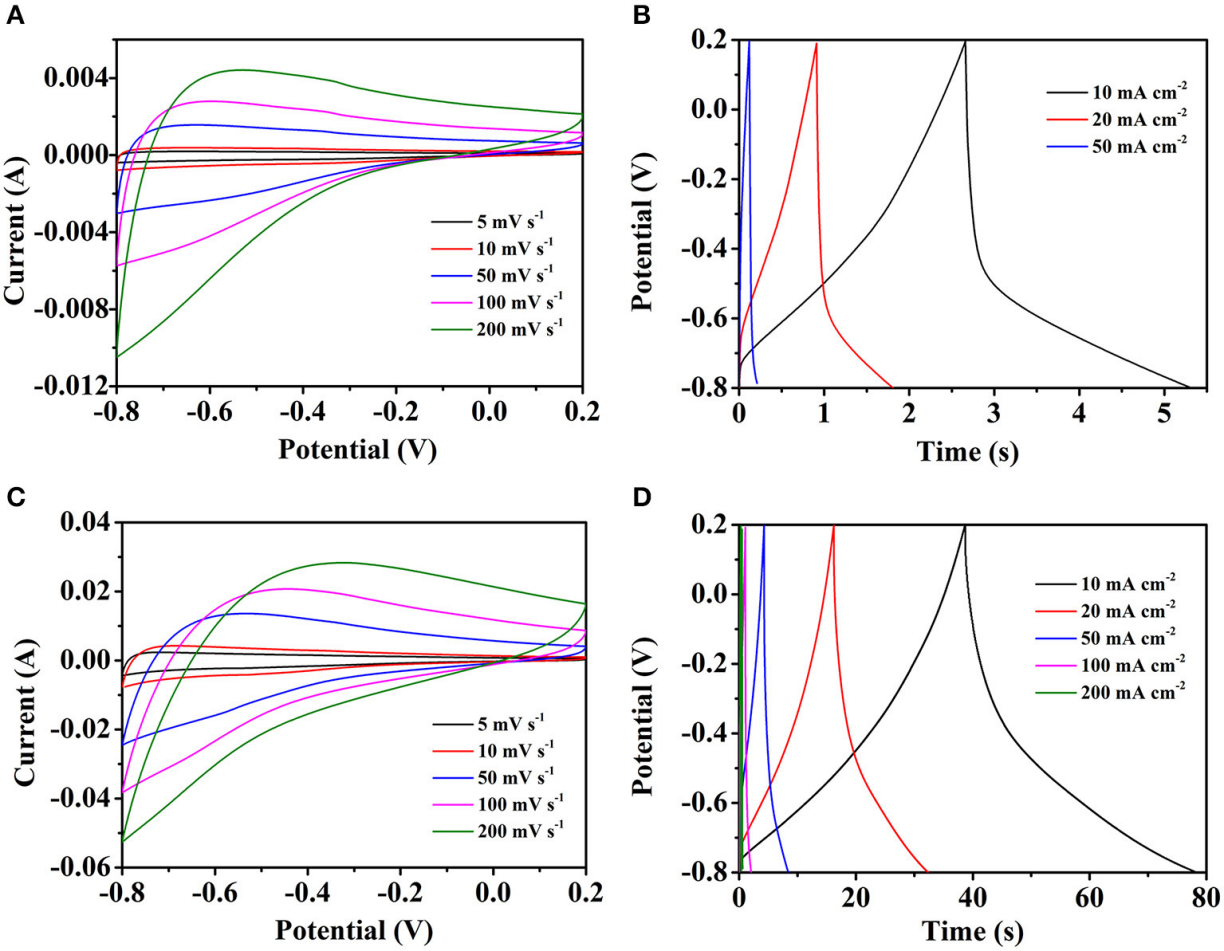
Representative CV curves of **(A)** carbon cloth and **(C)** Fe_2_O_3_/CC at different scan rate. The GCD curves of **(B)** carbon cloth and **(D)** Fe_2_O_3_/CC at different current densities.

To examine the electrochemical properties of the different electrodes, the CV, GCD and EIS were conducted in 3 M KOH aqueous solution electrolyte by the three-electrode test. Figures 5A,B shows the CV and GCD curves of pure carbon cloth, respectively. The slight redox peaks of the CV curves are probably caused by the oxygen functional groups which brought by the HNO_3_ clean process. The GCD curves also in accord with the CV curves. By the calculation, the specific capacitances are 30.7, 26.3, and 24.8 mF cm^−2^ at the current densities of 10, 20, and 50 mA cm^−2^, respectively. The electrochemical properties of the Fe_2_O_3_/CC electrode is also explored by the test. In Figure 5C, with the scan rate increasing, the shapes of the CV curves are similar, indicating the structure stability of the Fe_2_O_3_/CC. Because of the small load of Fe_2_O_3_ and the fast charge-discharge process, the redox peak cannot be found obviously. Typical GCD curves of the Fe_2_O_3_/CC at the current densities from 10 to 50 mA cm^−2^ are shown in Figure 5D. The curves are approximately symmetrical and linear, meaning the electrodes have outstanding electrochemical reversibility. By the calculation, the specific capacitances are respectively 422.2, 402.7, and 378.3 mF cm^−2^ at 10, 20, and 50 mA cm^−2^. Compared with the Fe_2_O_3_/CC electrode, the pure carbon cloth exhibits rather small capacitance, indicating the carbon cloth makes little contribute to the total capacitance of the Fe_2_O_3_/CC electrode. Besides, there are no obvious anodic/cathodic peaks appearing, which might be ascribed to that the porous Fe_2_O_3_ nanorodes are charged and discharged with a rapid Faradic reaction at a pseudo-constant rate (Chen et al., 2014).

**Figure 6:**
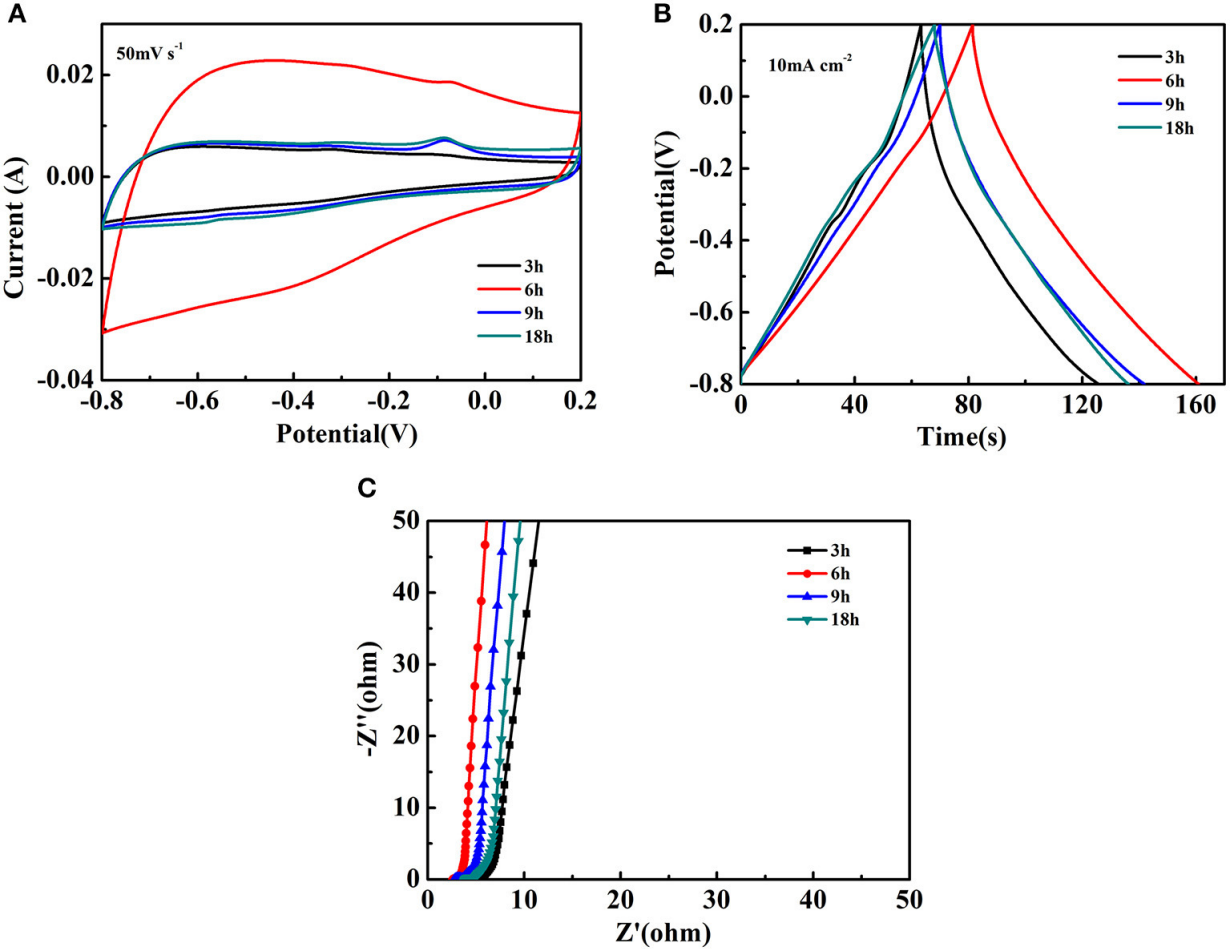
The electrochemical performance of Fe_2_O_3_@NiO/CC at different hydrothermal time: **(A)** the CV curves at the scan rate of 50 mV s^−1^, **(B)** the GCD curves at the current density of 10 mA cm^−2^, **(C)** the Nyquist plots.

The electrochemical studies for the Fe_2_O_3_@NiO/CC obtained by different hydrothermal time were also done by the three-electrode cell in 3 M KOH electrolyte. The CV curves of Fe_2_O_3_@NiO/CC are close to rectangular shape at the scan rate 50 mV s^−1^, which is an important characteristic in the supercapacitor showing the excellent electrical conductivity (Figure 6A). With the extended hydrothermal time, the core-branch Fe_2_O_3_@NiO on carbon cloth displays a pair of redox peaks. Compared with CV curves of Fe_2_O_3_/CC electrodes at the scan rate 50 mV s^−1^, the results indicates that the NiO realizes the electrochemical activity. Researches show that the anodic peak is caused by the oxidation of NiO to NiOOH, while the corresponding reverse process leads to generate of the cathodic peak, as shown in the following equation (Kong et al., 2011):

$$\begin{array}{l} {\text{NiO+OH}}^{-}↔\text{NiOOH+}\;{\text{e}}^{-} \end{array}$$

Because of the coefficient by carbon cloth and Fe_2_O_3_, the cathodic peak is not obvious.

Figure 6B illustrates the GCD curves of Fe_2_O_3_@NiO/CC between −0.8 and 0.2 V at the current density of 10 mA cm^−2^. The curves exhibits a predominantly symmetric nature and low IR drop, revealing the Fe_2_O_3_@NiO/CC nanostructures are good electrochemical capacitive characteristic with reversible redox reactions and small electrochemical impedance. The area specific capacitances of Fe_2_O_3_@NiO/CC derived from the discharge curves are 682.43, 816.13, 756.98, and 696.67 mF cm^−2^ for different hydrothermal time of 3, 6, 9, and 18 h, respectively. It is found that the area specific capacitances were enhanced with the extending the hydrothermal time from 3 to 6 h. However, further prolonging the time to 9 or 18 h, the capacitance decreased. In order to understand the inferior performance, the EIS study of Fe_2_O_3_@NiO/CC with different hydrothermal time is carried out (Figure 6C). The real axis intercept represents the equivalent series resistance (Meher and Rao, 2011; Meher et al., 2011). It is noteworthy that the equivalent series resistance of the Fe_2_O_3_@NiO/CC for 6 h is the minimum, which could be mainly attributed to the little synthetic quantity of NiO in 3 h and the increase in the thickness of the NiO layer from 6 to 18 h that hinders the diffusion and migration process of ions. Thus, the optimal experiment parameter can be determined to the hydrothermal time 6 h.

**Figure 7:**
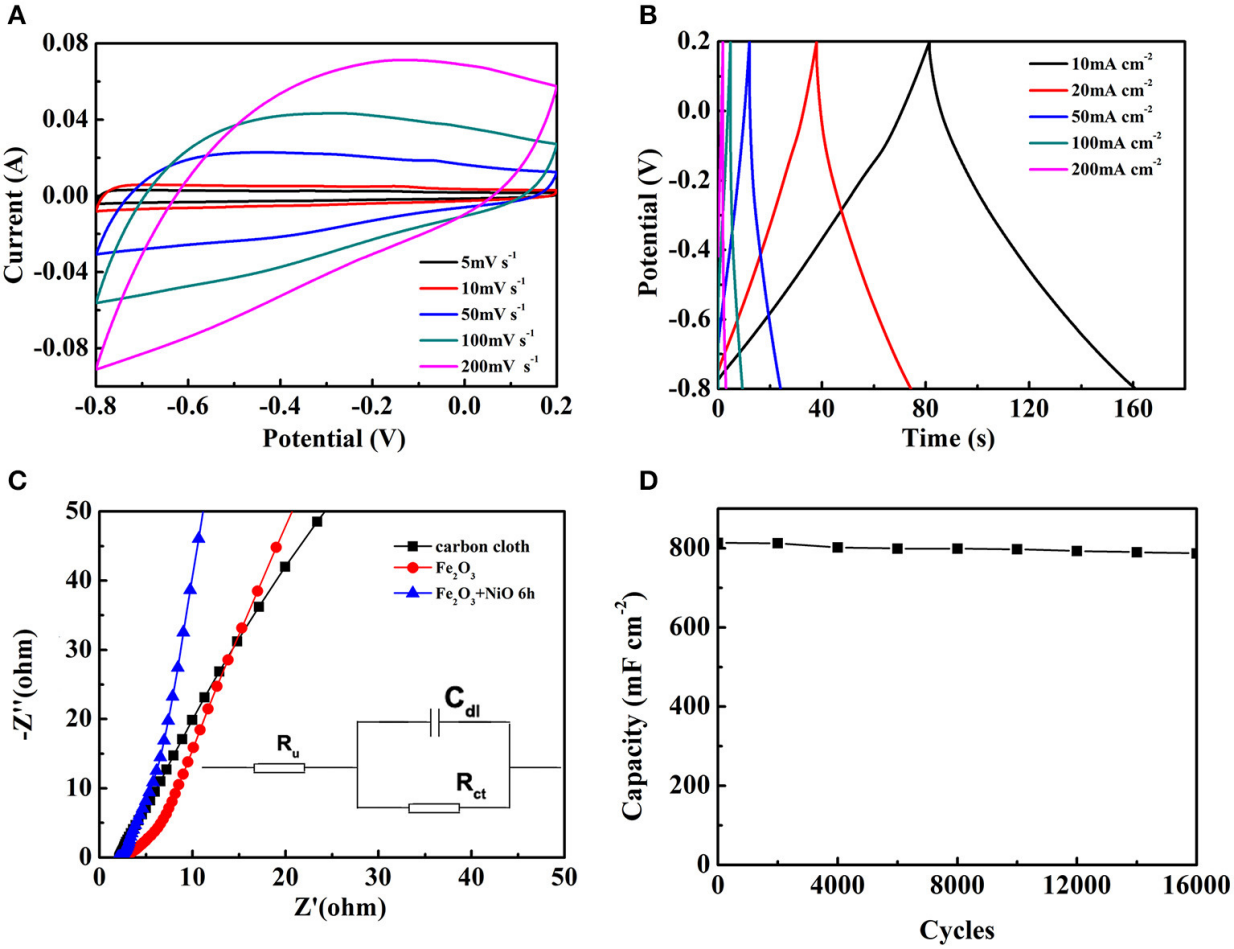
The electrochemical performance of Fe_2_O_3_@NiO/CC whose hydrothermal time is 6 h: **(A)** the CV curves at different scan rate, **(B)** the GCD curves at different current density. **(C)** The Nyquist plots of carbon cloth, Fe_2_O_3_/CC and Fe_2_O_3_@NiO/CC. **(D)** The long-term cycling performance of Fe_2_O_3_@NiO/CC.

To further explore electrochemical performance of the Fe_2_O_3_@NiO/CC with the optimum capacity, the CV and GCD tests of Fe_2_O_3_@NiO/CC obtained for 6 h hydrothermal process were performed (Figures 7A,B). As shown in Figure 7A, a series of CV curves of the optimal experiment parameter are collected at the scan rates from 5 to 200 mV s^−1^. With the scan rates increased, the shapes of the CV curves remain great and have little change, indicating a good kinetic reversibility of the Fe_2_O_3_@NiO/CC. The GCD curves for the optimal experiment parameter are nearly symmetric at 2–100 mA cm^−2^ (Figure 7B). The curves can be observed that the charge curves are symmetrical with their corresponding discharge curves, revealing that the electrode presents a great reversibility with a rapid response and small equivalent series resistance (Chen et al., 2013). Attributing to the high conductivity of carbon cloth and the porous feature of Fe_2_O_3_@NiO, the migration or diffusion of electron/ion can occur sufficiently during the rapid charge/discharge process (Fan et al., 2011). The areal capacitance are calculated by the charge-discharge curves (Figure 7B). The areal capacitance of Fe_2_O_3_@NiO are 849.54, 816.13, 768.29, 691.77, 542.33, and 490.12 mF cm^−2^ at current density of 5, 10, 20, 50, 100, and 200 mA cm^−2^, respectively. With the current density increasing, the areal capacitance is decreased. At high scan rates, the low areal capacitance is because the electrolyte ion movement and diffusion have be limited by the time constraint and only the outer active surface can be utilized for charge storage. On the other hand, the active surface area no matter outer or inner can be used for charge storage which thus leads to higher specific capacitance at lower scan rates (Wang et al., 2011). Those results directly reveal the advantage of Fe_2_O_3_@NiO for capacitance as electrode of supercapacitor.

In order to evaluate the contribution of NiO and Fe_2_O_3_ to the capacitance of the heterostructures, comparing the CV curves of Fe_2_O_3_@NiO/CC, Fe_2_O_3_/CC and pure carbon cloth (Figures 5A,C, 7A), the contribution of NiO and Fe_2_O_3_ to the capacitance of the heterostructures has been evaluated. The results indicate that the combination of NiO and Fe_2_O_3_ to a heterostructures can substantially enhance the electrochemical properties. Carbon cloth serves as current collector and substrate for deposition Fe_2_O_3_ nanorod, avoiding the addition of polymer binders/conducting additives and maintaining the robust mechanical stability. With the facile hydrothermal method, the Fe_2_O_3_ nanorods are directly grown on carbon cloth to ensure well mechanical adhesion, reducing the contact resistance between the active material and the current collector. Coating with branch-like NiO, the Fe_2_O_3_ nanorod material enhances its electrical conductivity, leading to a relatively small charge-transfer reaction resistance. It is beneficial for the rate capability of supercapacitors. Besides, the ultrathin NiO are incompletely wrapped on the surface of Fe_2_O_3_, which not only benefit for achieving fully electrochemical activity but also guarantee the core active material completely available to the ion in the electrolyte. The NiO also prevent the Fe_2_O_3_ nanorods from collapsing over long-time cycles in the electrolyte to improve the cycling stability. The excellent electrochemical performance of the supercapacitor device is owing to the good synergistic effect of branch-like NiO and Fe_2_O_3_ nanorods. Moreover, the porous feature of Fe_2_O_3_@NiO is beneficial for the electrons and ion transport more efficiently during the process of charge-discharge. It causes the specific capacitance have a large improvement. Those highlights have demonstrated the way to fabricate hybrid pseudocapacitive materials in exploiting the energy storage devices with outstanding electrochemical property.

In order to explore the internal resistance and the performance of electrode materials, EIS experiments were conducted (Figure 7C). In the Figure 7C, the diameter of the semicircle for the Fe_2_O_3_@NiO/CC electrode in the high frequency region is significantly smaller than that of Fe_2_O_3_/CC and pure carbon cloth, which illustrates the superior rate performance of the Fe_2_O_3_@NiO/CC electrode as well as implies that the Fe_2_O_3_@NiO/CC can reduce the contact and charge-transfer resistances in the electrode. The slope at low frequency part of Fe_2_O_3_@NiO/CC is also higher than that of Fe_2_O_3_/CC, demonstrating superior ion diffusion ability. To properly describe the action of an alternating potential input on supercapacitors, one can in principle consider at least two coupled interface processes influencing the impedance of the system: the electron transfer process across the electrolyte/electrode interface and the double-layer effect (Meher and Rao, 2011; Meher et al., 2011). The equivalent circuit typically has been schematized in the insert of Figure 7C, constituted by a solution resistance (R_u_), a charge transfer resistance (R_ct_) and a double-layer capacitance (C_dl_). R_u_ represents the uncompensated resistance of the electrolyte and other possible ohmic resistances, whereas R_ct_ represents the ohmic drop that can be associated to the electron transfer process. The double-layer defect, which roughly consists of charge separation in the electrode/electrolyte interphase as a result of charge migration, can be assimilated to a capacitor of capacitance C_dl_. The EIS further confirms the favorable performance of the Fe_2_O_3_@NiO/CC.

Cycling life test over 16,000 cycles for Fe_2_O_3_@NiO/CC was carried out at 20 mA cm^−2^ (Figure 7D). After 16,000 cycles of charge and discharge, Fe_2_O_3_@NiO/CC with 96.8 % capacitance retention shows better durability than the Fe_2_O_3_/CC (88.6 %). The morphology and structure change of Fe_2_O_3_@NiO/CC electrode after charge/discharge cycles were explored by SEM. Figure S3 demonstrates the Fe_2_O_3_@NiO/CC electrode after cycling for 16,000 cycles (as shown in Figure 7D). The structure is very similar to the morphology of pristine product (see Figures 2e,f). The stable cycling performance could be attributed to branch-like NiO which is covered on the surface of Fe_2_O_3_ and prevent the core part from collapsing during the reaction in long time (Guan et al., 2012). Besides, the synergistic effect between NiO and Fe_2_O_3_ could also make a contribution on the excellent cycling performance (Liu et al., 2011). The enhanced capacitance, rate capability and long cycling performance benefit from the improvement electrical conductivity after coating branch-like NiO. The high specific capacity and superior long-life cycle performance are much larger than those in previous works on Fe_2_O_3_/CC-based composites or nanostructured Fe_2_O_3_ (Jiao et al., 2014; Hu et al., 2015; Lu et al., 2015; Raut and Sankapal, 2016; Zheng et al., 2016; Zhang et al., 2017; Yang F. et al., 2018; Li F. et al., 2019).

## Conclusions

A novel and cost-efficient strategy has been developed to design a core-branch Fe_2_O_3_@NiO electrode active material on carbon cloth for flexible supercapacitor application. The Fe_2_O_3_@NiO nanorods directly grown on the carbon cloth can shorten the transfer paths of ions/electrons effectively, which reduces the contact resistance and avoid the use of polymer binders and conducting additives. The designed electrode exhibits a remarkably enhanced reversible capacity and cycling stability in comparison with Fe_2_O_3_ electrode, which should be ascribed to the fact that the ultrathin branch-like NiO incomplete coating on the surface of Fe_2_O_3_ nanorods. This study provides a novel strategy to construct high-performance flexible electrode materials with unique core-branch structure by incorporating two different pseudocapacitive materials.

## Data Availability Statement

All datasets generated for this study are included in the article/Supplementary Material.

## Author Contributions

MZ and NZ contributed conception and design of the study. MZ organized the database. MZ and XW performed the statistical analysis. MZ wrote the first draft of the manuscript. XL and DL wrote sections of the manuscript. All authors contributed to manuscript revision, read, and approved the submitted version.

### Conflict of Interest

The authors declare that the research was conducted in the absence of any commercial or financial relationships that could be construed as a potential conflict of interest.
