# Supplementary material for: Three-Dimensional Core-Branch α-Fe2O3@NiO/Carbon Cloth Heterostructured Electrodes for Flexible Supercapacitors
Source: Front Chem. 2020 Jan 8;7:887. doi: 10.3389/fchem.2019.00887 (PMC6960173; doi:10.3389/fchem.2019.00887)
Supplement: Supplementary file 1 [file Presentation_1.pdf]

Three-Dimensional Core-Branch α-Fe_2O_3@NiO/Carbon Cloth Heterostructured Electrodes for Flexible Supercapacitors

Miao Zhang[a, b, *], Xifei Li [a], Xiaohua Wang [b], Dejun Li [a], Naiqin Zhao [b, *]

[a] Tianjin International Joint Research Centre of Surface Technology for Energy Storage Materials, School of Physics and Materials Science, Tianjin Normal University, Tianjin 300387, China

[b] School of Materials Science and Engineering and Tianjin Key Laboratory of Composite and Functional Materials, Tianjin University, Tianjin 300072, China

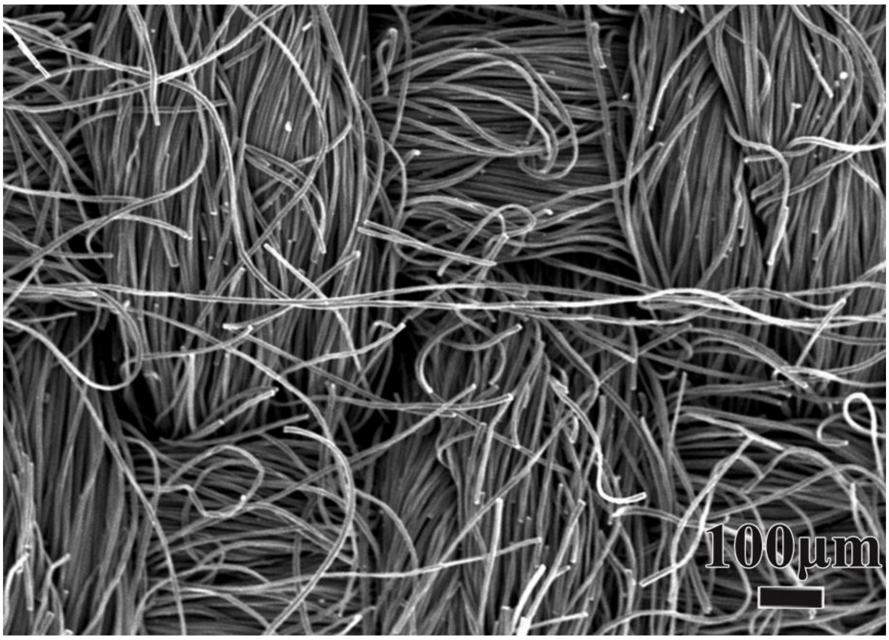

Fig. S1 The SEM image of the original carbon cloth

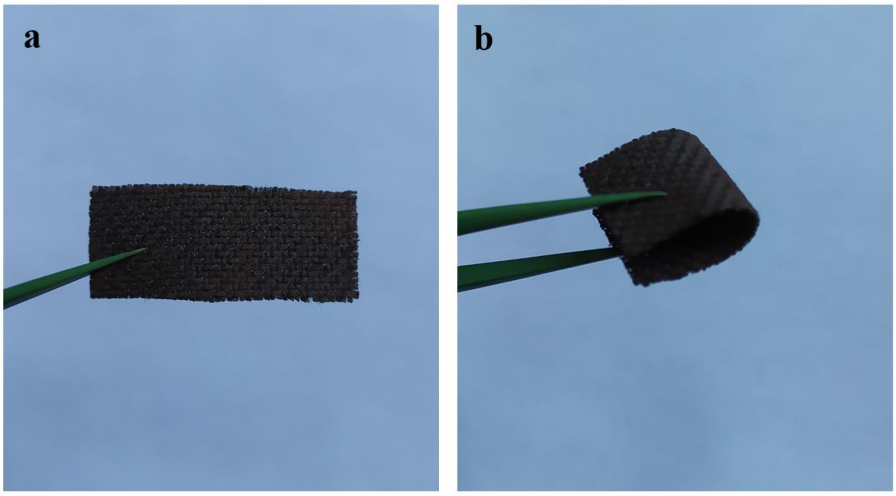

Fig. S2 The photo of the Fe_2O_3@NiO/CC (a) before and (b) after bending.

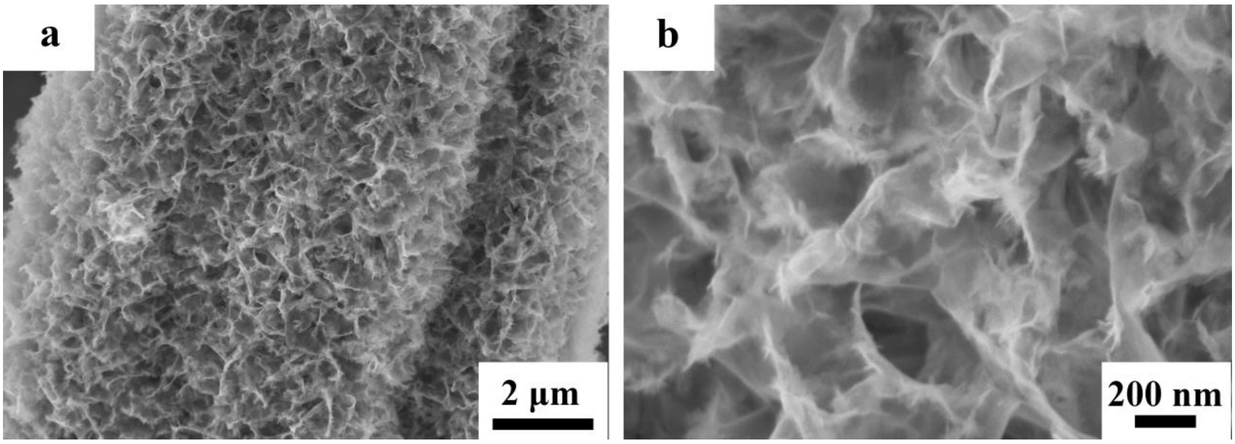

Fig. S3 Typical SEM image of the Fe_2O_3@NiO/CC after 4000 electrochemical cycles for the long-life cycle performance test in Fig. 7(d).
